# An Atypical Presentation of Sinonasal Tract Alveolar Rhabdomyosarcoma in a Young Male Patient Submitted to Multimodality Treatment

**DOI:** 10.1155/2021/8401755

**Published:** 2021-10-31

**Authors:** Wilber E. Bernaola-Paredes, Sergio Leonardo Favareto, Valdener Bella Filho, Eloah Pascuotte Filippetti, Walkiria Pantoja Bellotto, Henrique Rocha Mazorchi Veronese, Lucas Neves de Martins Moraes, Felipe D'Almeida Costa, Antonio Cassio Assis Pellizzon

**Affiliations:** ^1^Department of Radiation Oncology, A.C. Camargo Cancer Center, Sao Paulo, Brazil; ^2^Department of Stomatology, School of Dentistry, UNIFAMINAS, Minas Gerais, Brazil; ^3^Department of Anatomic Pathology, A.C. Camargo Cancer Center, Sao Paulo, Brazil

## Abstract

Rhabdomyosarcoma (RMS), a malignant tumor derived from the neoplastic proliferation of striated skeletal muscle cells, is the most common pediatric soft tissue sarcoma. Its treatment is mainly based on neoadjuvant chemotherapy (QT+), surgical resection, and adjuvant radiotherapy (RT+). RT+ has shown satisfactory results for locoregional control of the disease, in spite of promoting local side effects. The present case report was aimed at describing the clinical and therapeutic characteristics and the management of complications resulting from multimodal therapy in a patient with an atypical presentation of RMS in the sinonasal tract. A 20-year-old Afro-descendant man complained of an expansive tumor lesion, with left eye proptosis that reduced visual acuity and caused severe regional pain. Imaging analysis showed an extensive and infiltrative lesion in the periorbital region, sinonasal tract, left maxilla, and orbital roof. According to the histopathological analysis, the diagnosis was established corresponding to parameningeal alveolar RMS that was unresectable. Treatment was initiated with three cycles of QT+ which showed partial response and later RT+. After completing half of the RT+ sessions, the patient showed a complete response with reduction in tumor volume and improvement in pain and local conditions. Side effects such as alopecia and dermatological changes induced by radiation were observed. Moreover, painful erythematous areas were observed in the region of the hard and soft palate, uvula, and oropharynx, compatible with Grade 2 mucositis lesions. After the cytological swab test, some of them were diagnosed as herpes simplex lesions; thickening and decrease in salivary flow were also found. A local drug therapy approach was instituted, and photobiomodulation was performed to manage oral complications. RT+ was shown to be effective in locoregional control of the disease; however, the early management of its undesirable effects on the surrounding tissues was required.

## 1. Introduction

Sarcomas of the Head and Neck (SHN) are defined as malignant neoplasms derived from mesenchymal cells, whose origin is from bone, cartilage, muscle, vascular, adipose, and neurogenic tissues, with rapid growth, aggressive and infiltrative behavior, and the capacity for high locoregional recurrence [1, 2].

SHN are rare, representing fewer than 1% of all malignancies in this region. When they occur, establishment of their initial diagnosis and their proper management are challenging tasks, due to their location and the anatomical structures involved. Among the most common sarcomas, especially in the maxillofacial region, the most representative are rhabdomyosarcoma (RMS) and osteosarcoma (OS) of the gnathic bones [1, 2].

RMS is considered the most common soft tissue sarcoma in the childhood and in young patients, with an incidence of 3.5% in children under the age of 14 and approximately 2% in teenagers from 15-19 years of age, with 40% of the cases affecting the maxillofacial region, whereas the maxilla and mandible are the gnathic bones with the highest incidence among younger children [3, 4].

RMS is classified, according to the patient's age and the site affected, into 4 well-differentiated histological subtypes: (a) embryonic subtype, which has a better clinical prognosis and has a subdivision into botryoid and spine cells; (b) alveolar subtype, with an unfavorable prognosis; (c) undifferentiated subtype, also with an unfavorable prognosis; and (d) pleomorphic or anaplastic subtype, rarely found in children [3].

According to the primary location, RMS lesions are classified as parameningeal, nonparameningeal, and orbital. RMS of gnathic bones belong to the group of nonparameningeal entities and are mostly of the embryonic subtype (approximately 60% of the total reported cases) [3]. The diagnosis and classification of the lesion are established by immunohistochemistry assessment (IHC) exams and biomarkers; however, part of the cases remains unclassified, making their management difficult [1]. Among the sensitive and specific markers for RMS are Myogenin and MyoD1, both markers detected by IHC of initial muscle cell differentiation, which has a sensitivity of up to 100% [5].

The treatment of RMS is individualized, being mediated by the histological subtype of the tumor, its primary location and clinical staging, size, presence of free and/or microscopic and/or macroscopically compromised surgical margins (IRS-I, II, and III), distant metastases, and regional lymph node involvement, based on neoadjuvant chemotherapy (QT+), resective surgery, and/or adjuvant radiotherapy (RT+).

The role of RT+ in the treatment of RMS is still uncertain, but studies have demonstrated possible benefits in local control of the lesion, especially in the IRS II and III of any histological site and in the prevention of recurrence [2, 3]. However, side effects of RT+ occur on the surrounding tissues, such as regional radiodermatitis, skin peeling, itching, skin pigmentation and fibrosis, and alopecia and, in some cases and depending on the size of the irradiated field, damage to hearing has been observed [6, 7]. As regards the oral cavity, different degrees of mucositis in different anatomical sites, xerostomia, and hyposalivation, as well as opportunistic infections of candidiasis and herpetic and/or herpetiform lesions, whose etiology could also be associated with QT+, could be associated with RT+ as well [8].

Adjuvant therapies have been used to manage the side effects of multimodality therapy in order to decrease pain and general discomfort. Conventional medical management associated with photobiomodulation (laser therapy) and topical applications of corticosteroids have shown satisfactory clinical results in improving local conditions in affected patients [8].

The present case report is aimed at describing the clinical and therapeutic features of an atypical RMS of the sinonasal tract in a young patient who underwent multimodal therapy and management of local side effects.

## 2. Case Report

**Figure 1:**
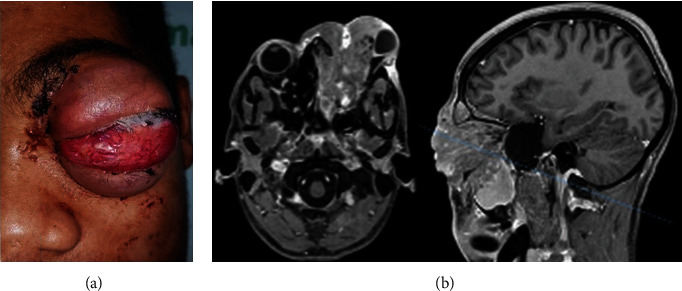
Clinical and imaging features. (a) Extraoral examination showed exophytic and expansive lesion around orbital region. (b) MRI axial and sagittal slices demonstrated tumor invasiveness into the periorbital region, sinonasal tract, left maxilla, and orbit roof.

A 20-year-old Afro-descendant man, complained of a painful, exophytic, and expansive lesion, with rapid growth in the left hemimaxilla region, affecting the orbital region, with ipsilateral ocular proptosis and partial loss of visual acuity (Figure 1(a)). The Magnetic Resonance Imaging (MRI) exam revealed an expansive and infiltrative lesion centered in the maxillary sinus/left osteomeatal complex, extending to the periorbital and orbital region with left proptosis and extra-axial intracranial invasion that measured 91 × 75 × 57 mm (Figure 1(b)).

**Figure 2:**
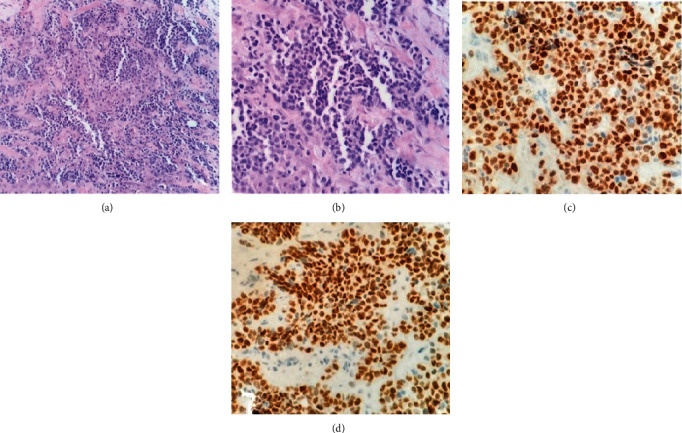
Histological and IHC analysis of alveolar RMS. Small cells with scarce cytoplasm and rounded and hyperchromatic nucleus grouping into nests with pseudoalveolar spaces under optical microscopy at 20x (a) and 40x (b). Strong and diffuse nuclear marking (higher than 50%) of myogeny (c) and MyoD1 (d) in neoplastic cells that corroborated initial diagnosis.

Histopathological analysis was performed by incisional biopsy of the lesion in the supraorbital region, in the submental lymph node, and in the nasal region (Figure 2), the results of which confirmed its undifferentiated mesenchymal origin. Moreover, based on the immunohistochemistry (IHC) assessment, the final diagnosis of alveolar parameningeal RMS was established, corroborated by clinical, histological, and imaging data.

**Figure 3:**
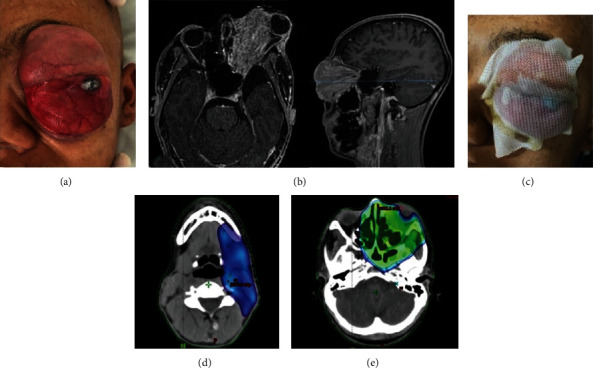
Clinical examination after neoadjuvant QT. (a) Partial response to treatment was observed with increased tumor volume. (b) MRI in axial and sagittal slices confirmed. (c) CT for RT planning. (d) PTV contouring included submental lymph nodes. (e) CTV for tumor irradiation.

Initial management was based on neoadjuvant chemotherapy in order to avoid distant metastasis and achieve reduction in the tumor volume. Three initial cycles of QT+ were performed, based on ifosfamide (1800 mg) and etoposide (100 mg) that showed a partial response observed clinically (Figure 3(a)) and in the imaging examination (Figure 3(b)).

RT concomitant with QT (protocol based on vincristine 2 mg + ifosfamide 1.8 g/m^2^-04 more cycles) was established after no initial response. Computed Tomography (CT) was performed for initial RT planning (Figure 3(c)), and no metastasizing lesions were observed. The Intensity Modulated Radiotherapy (IMRT) technique was used to deliver a dose of 2 Gy/day of 50 Gy in the area of the prechemotherapy tumor and positive submental lymph node (Figure 3(d)); and 60 Gy was delivered in the area of the postchemotherapy tumor (Figure 3(e)).

**Figure 4:**
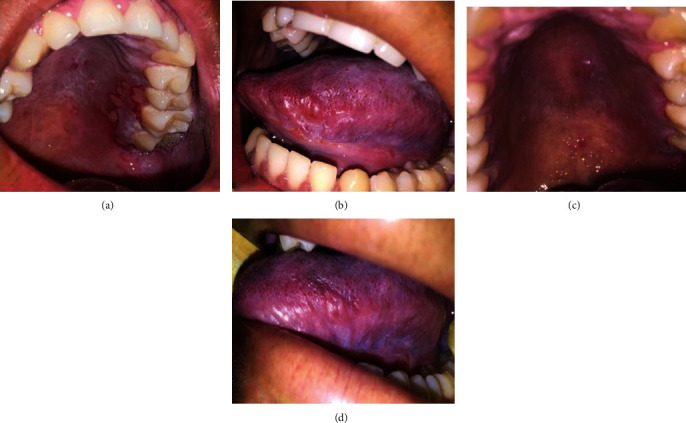
Oral manifestations resulting from radiotherapy and therapeutic evolution of complications. Herpes simplex and Grade 2 mucositis lesions shown in region of the hard palate (a) and in the oropharynx and left lateral border of the tongue (b), respectively. Intraoral clinical appearance after local drug therapy and photobiomodulation (PBM), with regression of lesions in the hard palate (c), oropharynx, and left lateral border of the tongue (d).

During RT, painful erythematous areas were observed in the hard and soft palate associated with herpes simplex lesions confirmed by exfoliative cytology, mucositis lesions on the lateral edge of the tongue (Figures 4(a) and 4(b)), and decreased salivary flow. Local control of symptoms was instituted with drug therapy, based on the topical use of Mylanta Plus 5 ml for 15 days, 03 times a day; Nystatin (100,000 IU) 5 ml, 3 times daily, remaining in the mouth for 4 minutes every time it was used, for 07 days; and acyclovir 200 mg every 4 hours for 07 days. Photobiomodulation (laser therapy) was performed for improving the mucositis lesions, by using diode laser (Therapy EC, DMC, São Carlos, São Paulo, Brazil) with a beam output power of 100 mW, 4 J per point across the affected region (04 points per region), and a satisfactory clinical response was observed (Figures 4(c) and 4(d)).

**Figure 5:**
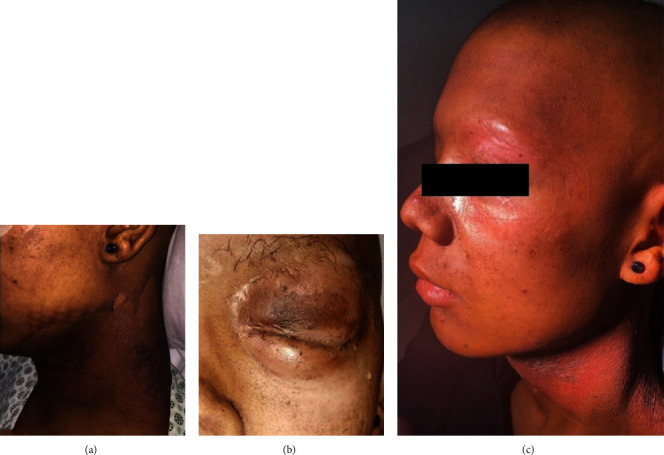
Extraoral manifestations after RT. Dermatological changes induced by radiation (a) in the periorbital region (b) and local alopecia (b, c). (c) Clinical appearance after local use of ointments and moisturizing creams.

In addition, areas of radiation-induced dermatitis were visualized in the left neck (Figure 5(a)) and in the ipsilateral periorbital region (Figure 5(b)); and alopecia (Figures 5(b) and 5(c)) was observed. The daily use of ointments and moisturizing creams was indicated for improvement of local condition (Figure 5(c)).

**Figure 6:**
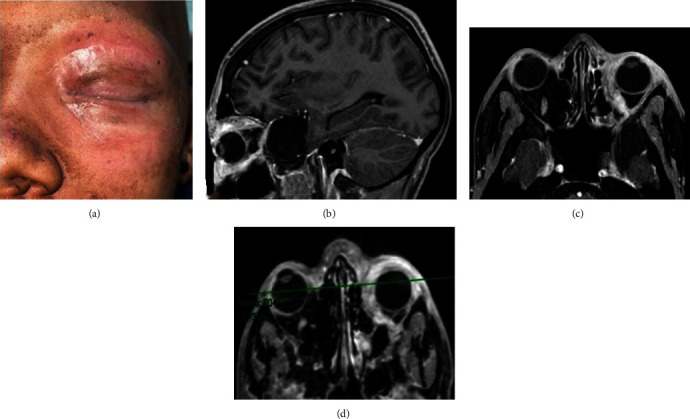
Clinical and imaging analyses after final RT session. (a) Reduced tumor volume was observed and by MRI in sagittal (b) and axial (c, d) slices that showed complete response to treatment with LCR obtained.

At the final RT session, local tumor control was observed (Figure 6(a)). On MRI examination at 60 days after RT, decreased tumor volume was obtained (Figures 6(b)–6(d)); however, two months later, during a new cycle of adjuvant QT performed in January 2021 and based on vincristine (2 mg/IV), a metastasized lesion to the brain caused the death of the patient.

## 3. Discussion

RMS is the most common soft tissue sarcoma among children, accounting for 51% of childhood soft tissue SHN [4]. The international classification of pediatric sarcomas considers that the alveolar subtype has the worst prognosis, similar to that of undifferentiated sarcoma that affects slightly older patients, at a mean age ranging between 14 and 19 years of age [9].

In the present case, an atypical lesion in the sinonasal tract was diagnosed by histopathological analysis, based on the presence of small cells with scarce cytoplasm and rounded and hyperchromatic nuclei that formed nests with pseudoalveolar spaces, with adhesion to the walls, sometimes forming aspects of a tombstone. Moreover, morphological findings and sensitive, specific biomarkers for RMS, Myogenin and MyoD1, were used. Both are IHC markers of initial muscle cell differentiation, and sensitivity of up to 100% has been reported in studies [5]. The main differential diagnosis of the present case was the embryonic subtype since it can morphologically mimic the alveolar subtype. However, the diffuse nuclear expression of Myogenin and MyoD1 (higher than 50% in cells) strongly favored the diagnosis of the alveolar subtype, since the embryonic subtype has nuclear marking of less than 50%, with an average of 1-10% of the cells [10].

Multimodal therapy has been used in the treatment of RMS, and these treatments contribute to locoregional control (LCR) of the disease, especially in those where the surgical approach is restricted due to the extension and impairment of anatomic structures [1, 3]. In this case report, a favorable clinical response to LCR was shown, based on neoadjuvant QT+ followed by concomitant RT+.

Management of these complications caused by multimodality treatment is essential in order to improve the quality of life and to maintain basic functions such as deglutition, chewing, and phonation, and there are numerous approaches with satisfactory results reported in the literature [3, 7]. Although RT+ techniques have improved with reference to dose delivery restricted to tumor volume and by reducing the spread of the dose to surrounding tissues, nevertheless, undesirable local side effects are still observed [6].

For the management of skin lesions, some adjuvant therapies such as phototherapy, photobiomodulation (FBM), and topical drug applications help in the treatment of these complications [3]. In addition, for lesions in the oral cavity, topical medications based on hydrogels, antiseptics containing chlorhexidine, ointments, and PBM have been used [7]. For this case, RT by the IMRT technique, better LCR of the disease was achieved with a decrease in tumor volume; however, side effects were observed at the site, mainly at the level of the oral cavity and oropharynx. The management of oral complications with topical drug therapy using antifungal solutions and systemic antiviral medication in association with PBM sessions showed satisfactory clinical results of RT.

## 4. Conclusion

RMS of the sinonasal tract is rare, but LCR by multimodal therapy could be achieved. Side effects associated with treatment must be managed and reduced with the use of combinations of adjuvant therapies with palliative intent and pain control. Prospective studies should be conducted in order to gain better understanding of the role of each therapy in the multimodal management.
